# Single layer centrifugation combined with brief abstinence for semen cryopreservation in a patient with hematospermia: Case report and literature review

**DOI:** 10.1097/MD.0000000000043272

**Published:** 2025-07-18

**Authors:** Yuan Liu, Yang Xian, Bo Liu, Wenrui Zhao, Lijuan Ying, Jinyan Xu, Xuefeng Luo, Chen Luo, Fuping Li

**Affiliations:** a Department of Andrology/Human Sperm Bank of Sichuan Province, West China Second University Hospital, Sichuan University, Chengdu, Sichuan, P.R. China; b Key Laboratory of Birth Defects and Related Diseases of Women and Children (Sichuan University), Ministry of Education, Chengdu, Sichuan, P.R. China.

**Keywords:** ejaculatory duct cyst, hematospermia, single layer centrifugation, sperm cryopreservation

## Abstract

**Rationale:**

Efficient semen preservation is important in patients with hematospermia (HS). Few reports have delineated effective methods for the removal of red blood cells (RBCs), and existing techniques have exhibited constraints that affect sperm preservation. There is a paucity of studies on semen preservation in HS patients.

**Patient concerns:**

Prior to surgical intervention for an ejaculatory duct cyst, the patient expressed concerns regarding potential fertility impairment. Considering his intention to conceive in the near future, he opted for sperm cryopreservation in a sperm bank as a precautionary measure.

**Diagnoses:**

We report the case of a patient who presented with HS and was diagnosed with an ejaculatory duct cyst accompanied by cyst wall calcification. Semen analysis revealed the presence of numerous RBCs in the ejaculate.

**Interventions:**

Three semen processing techniques to eliminate blood contamination were evaluated in the laboratory. (A) Processing of the RBC lysis buffer, (B) density gradient centrifugation, and (C) single layer centrifugation (SLC). Donor semen mixed with peripheral blood was used for the experiment. Post-thawing progressive motility (%) of the sperm was evaluated using computer-assisted sperm analysis. Additionally, the patient was advised to undergo a brief period of abstinence (4 hours) to diminish the quantity of RBCs in the semen and to facilitate the freezing of additional straws.

**Outcomes:**

The SLC can effectively remove RBCs from semen while maintaining high sperm recovery and post-thawing progressive motility. The combination of SLC and very short-term abstinence elevates the quantity of frozen sperm relative to alternative compositions. Ultimately, the patient successfully cryopreserved 5 semen straws.

**Lessons:**

Before commencing formal treatment, it is essential to evaluate fertility preservation in individuals with HS. Employing SLC and subsequent semen collection following a brief abstinence period can mitigate the adverse effects of physical damage on sperm and avert the influence of RBC lysis on the efficacy of sperm cryopreservation. This method offers an effective strategy for autologous semen cryopreservation in a clinical setting for patients with HS.

## 1. Introduction

Hematospermia (HS), commonly referred to as the presence of blood in semen, has a multifaceted etiology, including infectious diseases, nonspecific inflammation, and reproductive system cancer.^[1]^ Although HS is often benign and short-lived, it can pose significant challenges for certain patient groups, such as those intending to conceive in the near future, individuals experiencing reduced fertility due to HS, and patients with tumors who require fertility preservation before undergoing iatrogenic interventions such as radiotherapy. Consequently, sperm cryopreservation from HS patients demands special attention in clinical laboratories.

At present, there is a notable deficiency in studies on cryopreservation of human bloody semen.^[2]^ Hemoglobin released from lysed red blood cells (RBCs) is an essential source of reactive oxygen species, which can lead to sperm dysfunction and diminish the efficiency of sperm cryopreservation.^[3]^ Therefore, appropriate treatment and preservation of HS are critical to reduce these detrimental effects. Conventional RBC lysis treatments have been reported to affect sperm motility adversely.^[4]^ Colloid centrifugation is a prevalent technique for eliminating undesirable components such as bacteria, viruses, and white blood cells (WBCs), and it is crucial in the selection of human and animal sperm.^[5,6]^

Our study reports the effective cryopreservation of sperm from a patient with HS. Simulation and verification showed that single layer centrifugation (SLC) and brief abstinence (2–5 hours) before sperm collection can effectively remove RBCs from semen while preserving a high recovery rate of sperm motility. These findings present a useful approach for cryopreservation of HS, potentially improving outcomes for individuals requiring fertility preservation.

## 2. Materials and methods

### 2.1. Sperm motility parameter analysis

Semen was collected from the patients and volunteers through masturbation. Samples were incubated at 37 °C in a constant-temperature shaker until liquefaction was achieved. A 7 μL of semen was then added to the Makler counting chamber (Sefi Medical Instruments). Sperm were evaluated in accordance with the 6th edition of the World Health Organization (WHO) Laboratory Manual for the Examination and Processing of Human Semen, and sperm motility parameters were analyzed using a computer-assisted sperm analysis (CASA) (SSA-Ⅱ; Suiplus, Shanghai, China).^[7]^ The motility of sperm pre- and post-thawing was determined by straight-line swimming velocity (VSL) value, categorized as grade A (rapid progressive, 25 μm/s ≤ VSL), grade B (slow progressive, 5 ≤ VSL < 25 μm/s), and grade C (nonprogressive, 0 ≤ VSL < 5 μm/s).

### 2.2. Counting RBCs in semen

After adding semen to the Makler counting chamber, the concentration of RBCs was determined by counting all the cells within the central 100 squares of the counting slide. Each sample was counted twice, and the average was taken.

### 2.3. The removal of RBCs and sperm cryopreservation

**Figure 1.**
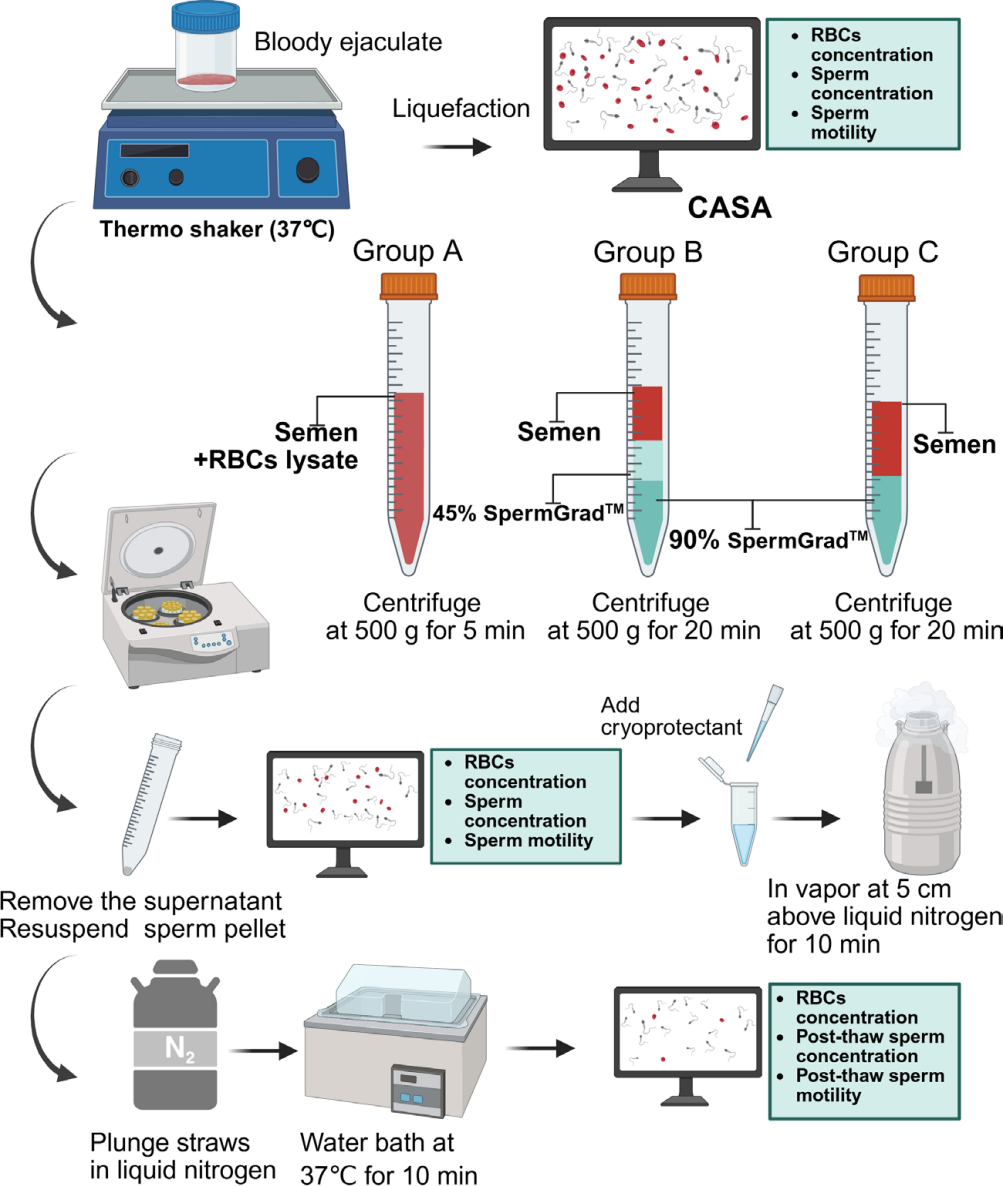
Schematic illustration for the removal of red blood cells (RBCs) in semen. The RBCs in the samples were removed by 3 different methods: RBC lysis buffer treatment, density gradient centrifugation (DGC), and single layer centrifugation (SLC). After freezing and thawing, sperm concentration and motility parameters were measured by computer-assisted sperm analysis (CASA). The figure was created with BioRender.

Healthy volunteers were recruited from the Sichuan Human Sperm Bank, and semen and blood were mixed to simulate HS formation. The experimental groups were delineated according to the treatment protocols (Fig. 1): Group A: semen was mixed with RBCs lysis buffer (Solarbio, Beijing, China) and added to a 15 mL centrifuge tube (Falcon, USA) with the semen and lysed for 5 minutes, followed by centrifugation at 500 × *g* for 5 minutes, and the supernatant was discarded after centrifugation (5430R; Eppendorf, Germany). Group B: 1 mL of 90% and 1 mL of 45% SpermGrad™ (Vitrolife, Sweden AB) was diluted using IVF sperm medium (10,136; G-IVFTM PLUS, Vitrolife, Sweden) and successively layered at the bottom of a centrifuge tube, followed by the addition of semen on top, and centrifugation at 500 × *g* for 20 minutes. Group C: A 2 mL aliquot of 90% SpermGrad™ was introduced to the base of the centrifuge tube, followed by the addition of semen and subsequent centrifugation at 500 × *g* for 20 minutes. Following the described 3 procedures, the precipitate was resuspended in 500 μL IVF sperm medium. A 10 μL aliquot was added to the Makler counting chamber, and sperm concentration and progressive motility (PR) were assessed using CASA. Cryoprotectants without animal components (1:4) were subsequently included in the IVF sperm medium.^[8]^ Each straw (010288; Cryo Bio System, Paris, France) contained 100 μL of the prepared suspension and was positioned in vapor 5 cm above liquid nitrogen for 10 minutes before being plunged into liquid nitrogen. After thawing in a 37 °C water bath for 10 minutes, the quantities of RBCs and sperm, together with post-thawing sperm motility, were assessed using CASA.

## 3. Case presentation

### 3.1. Medical history

A 27-year-old male software engineer was diagnosed with HS. He pursued medical evaluation because of blood in his semen, and an ultrasound examination identified an ejaculatory duct cyst with calcification of the cyst wall. The patient was informed of the potential risks of impaired fertility associated with surgery and expressed a need for forthcoming reproductive plans. The patient will proceed to a sperm bank for sperm cryopreservation. The patient denied any history of erectile dysfunction. His medical records revealed no documented diagnoses of diabetes mellitus, cardiovascular disease, or hypertension, nor was he currently receiving pharmacological interventions for systemic medical conditions. Ultrasound testing showed normally positioned bilateral testes with symmetrical testicular volumes of 12 mL.

**Figure 2.**
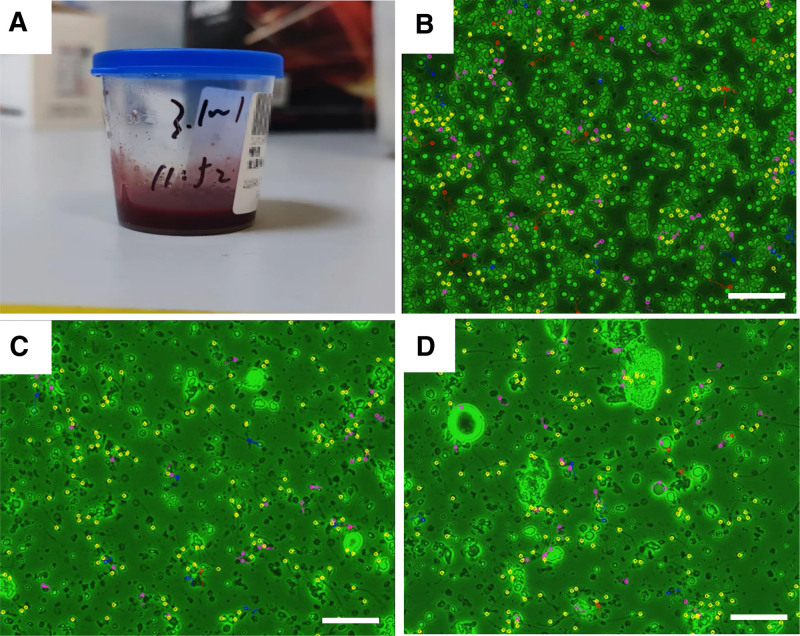
The computer-assisted sperm analysis (CASA) results for the patient with hematospermia. (A and B) The semen from the patient with hematospermia appears bright red, and a high concentration of red blood cells is observed under microscopy. (C and D) Pre- and post-thawing sperm motility parameters were determined using CASA. Sperm motility levels are shown with different colors: red (grade A), blue (grade B), purple (immobile sperm), and yellow (grade D). The scale bar represents 50 μm.

The semen analysis indicated a volume of 3.1 mL and bright red coloration of the semen (Fig. 2A). Microscopic enumeration revealed an RBC concentration of 835 × 10^6^/mL, sperm concentration of 63 × 10^6^/mL, and PR of 46% (Fig. 2B). Pre-freezing infectious pathogen examinations, including serological screening for human immunodeficiency virus, syphilis, hepatitis B, and hepatitis C viruses, were conducted to guarantee the safety of the cryopreservation procedure. Additionally, bacterial and mycoplasma cultures were performed on semen samples. All the pathogen tests yielded negative results.

### 3.2. Laboratory research

To efficiently eliminate RBCs and prepare sperm for cryopreservation, the patient’s residual semen underwent routine RBC lysis using lysis buffer. The initial lysis treatment lasted for 3 minutes, and microscopic examination revealed a considerable quantity of RBC debris (Fig. 2C). An additional 2 minutes of lysis was administered. The prepared sperm was subsequently subjected to freezing attempts. The post-thawing PR was 5%, the motility was 11%, and the recovery rate fell short of the expected levels (Fig. 2D).

**Figure 3.**
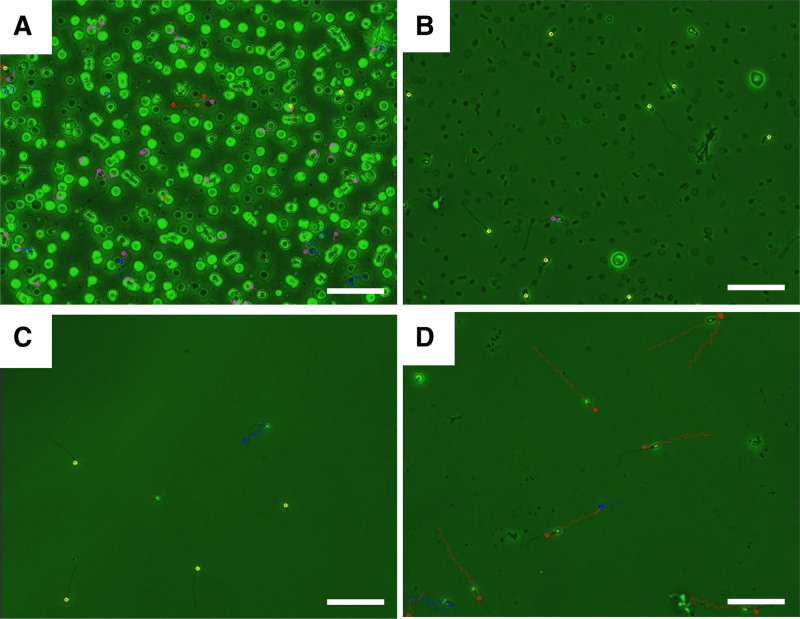
Comparison of motile sperm recovery across 3 experimental treatments for hematospermia. (A) Red blood cells were incubated with semen to simulate hematospermia formation. Computer-assisted sperm analysis (CASA) results for (B) red blood cell (RBC) lysis treatment, (C) density gradient centrifugation (DGC), and (D) single layer centrifugation. Sperm motility levels are shown with different colors: red (grade A), blue (grade B), purple (grade C), and yellow (immobile sperm). The scale bar represents 50 μm.

The 3 RBC removal methods described above were implemented to determine a better laboratory protocol for HS treatment (Fig. 1). A blood/sperm admixture from volunteers was used to simulate HS (Fig. 3A). The RBC concentration in the semen was adjusted to 527 × 10^6^/mL, and the CASA results showed a sperm concentration of 31 × 10^6^/mL and PR of 51%. Preexperimental results indicated that Group A exhibited a large amount of RBC debris residue and low PR of sperm (Fig. 3B), whereas Group B showed a substantial decline in motile sperm (Fig. 3C). In contrast, Group C demonstrated superior retention of sperm concentration and PR (Fig. 3D).

### 3.3. Cryopreservation outcome of HS

**Figure 4.**
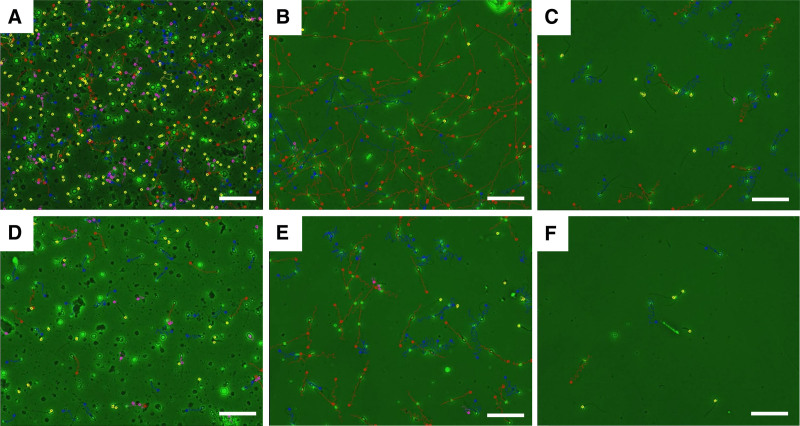
Assessment of red blood cell removal and sperm cryopreservation efficacy in hematospermia. (A) In the semen sample obtained during the first ejaculation, microscopic examination revealed a large number of red blood cells (RBCs) and their fragments. (B and C) Sperm parameters after single layer centrifugation and post-thawing motility were determined using computer-assisted sperm analysis (CASA). (D) After 4 hours of abstinence, the sperm and RBC concentrations in the semen of patients decreased. (E and F) CASA results for the second freezing sperm parameters for SLC and post-thawing motility. Sperm motility levels are shown with different colors: red (grade A), blue (grade B), purple (grade C), and yellow (immobile sperm). The scale bar represents 50 μm.

The patient revisited the sperm bank for sperm cryopreservation after 7 days, and the volume of semen obtained was 2.6 mL. Microscopically, it showed stale hemorrhagic RBC, with a concentration of 95 × 10^6^/mL, a sperm concentration of 137 × 10^6^/mL, and a PR of 34%. Following the preexperimental results, we conducted SLC, employing IVF sperm medium for dilution and including cryoprotectants. We successfully froze 3 straws with a freezing volume of 100 μL. The straws were sealed and cooled above liquid nitrogen for 10 minutes before storage in liquid nitrogen. The motility after thawing was 70% (Fig. 4A–C).

Considering the adverse effects of ruptured RBCs on sperm viability and the patient’s forthcoming surgery, a brief period of abstinence is recommended to increase the quantity of frozen sperm. The subsequent semen sample, obtained 4 hours later, had a volume of 1.7 mL with an RBC concentration of 56 × 10^6^/mL, a sperm concentration of 85 × 10^6^/mL, and PR of 45%. Following SLC for semen, 2 straws of semen samples were cryopreserved, resulting in a post-thawing sperm concentration of 9.0 × 10^6^/mL, and PR of 45% (Fig. 4D–F). Five straws were successfully frozen for the patient on the same day for future utilization (Table 1).

**Table 1:** The parameters of semen.

	Abstinence interval	V (mL)	Color	Sperm C (×10^6^/mL)	Sperm PR (%)	RBC C (×10^6^/mL)	Sperm suspension V (mL)	Sperm C after SLC (×10^6^/mL)	Sperm PR after SLC (%)	Progressive sperm recovery rate (%)		Post-thawing sperm C (×10^6^/mL)	Post-thawing sperm PR (%)	Freezing V (mL)	Straws
Semen examination before freezing	5 days	3.1	Bright red	63	46	835	0.5	–	–		5	–	–	–	–
1st semen cryopreservation	7 days	2.6	Dark red	137	34	95	0.15	112	90		24	37	70	0.1	3
2nd semen cryopreservation	4 hours	1.7	Dark red	85	45	56	0.15	54	88		25	9	45	0.1	2

C = concentration, PR = progressive motility (%), RBC = red blood cell, SLC = single layer centrifugation, V = volume.

## 4. Discussion

With the advancement of cryogenic technology, fertility preservation in individuals facing disease-induced fertility reduction has garnered increasing attention. Cryopreservation techniques such as oligospermia or single sperm cryopreservation have provided significant support to populations with severely diminished fertility.^[9]^ Invasive procedures such as testicular sperm extraction (TESE) and microdissection TESE have also emerged as viable options for achieving successful progeny.^[10]^ Nonetheless, morphological abnormalities, the presence of infectious pathogens, and a heterogeneous semen environment necessitate the optimization of processing and cryopreservation protocols tailored to specific clinical scenarios and patient needs.

### 4.1. Etiologies of HS and their effect on sperm function

Patients with HS are typically characterized by a high concentration of RBCs in their semen, with most cases being benign and self-limiting.^[11]^ The most common medical cause of HS is transrectal ultrasound-guided prostate biopsy, while infections and nonspecific inflammation, particularly of the prostate or seminal vesicles, are also prevalent.^[12]^ Although oncological morbidity associated with HS is minimal, testing the amount and duration of ejaculatory bleeding is essential. Recurrent and persistent bleeding in patients with HS can lead to sexual dysfunction attributable to worry and apprehension about sexual activity.^[13,14]^ Treatment options, including fluoroscopic-assisted transurethral incision of the ejaculatory ducts and holmium laser incision via ureteroscope, may lead to complications such as retrograde ejaculation and obstructive azoospermia. Additionally, these procedures may induce psychological distress for the patient’s partner due to the presence of HS.^[1]^ Cryopreservation of sperm in patients with HS is a viable technique for maintaining cryobiological characteristics and possesses notable clinical importance.

Previous studies have reported that an abnormal state of peripheral blood cells causes a decrease in male fertility.^[15]^ Semen is comprised of sperm, seminal plasma, immune cells, and epithelial cells, all of which significantly influence sperm activity. Contamination by bacterial, viral, and other cellular components, such as the accumulation of RBCs in semen resulting from various diseases, can negatively affect sperm function.^[16]^ Oxygen free radicals released from lysed RBCs are detrimental to sperm, and the dissociation of iron ions can react with the sperm membrane surface, leading to lipid peroxidation.^[17]^ The presence of blood in semen can also stimulate the production of antisperm antibodies, resulting in sperm aggregation and reduced fertility.^[18]^ Moreover, HS is a potential sign for sexually transmitted infectious pathogens, such as herpes simplex virus, *Chlamydia trachomatis*, and *Ureaplasma urealyticum*.^[1,19]^

### 4.2. RBC lysis buffer in HS

Normal seminal plasma is crucial for maintaining sperm motility and facilitating fertilization in the female reproductive tract.^[20]^ Disease conditions, including viral and bacterial infections, can increase the concentration of inflammatory factors in seminal fluid.^[21]^ An increase in WBCs, especially macrophages, can produce oxygen free radicals and elevate the sperm DNA fragmentation index (DFI), which is detrimental to sperm motility and embryonic development.^[22]^

RBC lysis buffer is routinely employed in cellular experiments to eliminate RBC resulting from surgical residues or contamination. Yazdinejad et al conducted a study demonstrating that the erythrocyte lysing buffer reduced testicular sperm viability and significantly elevated DFI.^[4]^ The negative effects on sperm are attributed to the presence of band 3 anion channels on the sperm surface, which are toxic to sperm survival.^[23]^ Additionally, the RBC lysis buffer can induce osmotic shock, affecting sperm viability by disrupting metabolism.^[24,25]^ Sperm are particularly vulnerable to oxidative stress due to insufficient antioxidant levels, which can result in DNA damage and potentially lead to miscarriage or complex diseases in offspring.^[26]^ Lysed RBCs produce oxygen free radicals, and recent research indicates that high levels of reactive oxygen species are the primary cause of cryoinjury.^[27]^ Soygur et al simulated a testicular sperm scenario using an erythrocyte-sperm separation medium, which did not adversely affect sperm morphology and viability, improved the efficiency, and shortened the time of sperm retrieval.^[28]^ Nevertheless, the handling of RBCs in that study differed significantly from clinical practice.^[4]^ Ammonium chloride, a key component of RBC lysis buffer, has been shown to have adverse effects on both DNA and embryos.^[29]^ Current sperm analysis measures, including changes in morphology and the extent of DNA damage, emphasize the necessity for reliable and cautious clinical laboratory operations.

### 4.3. Removal of non-sperm components from semen

The removal of urine from semen is straightforward through centrifugation, but cellular contaminants such as RBCs and WBCs, are of greater concern. Recent studies have proposed magnetic-activated cell sorting to eliminate contaminating leukocytes and erythrocytes from TESE samples,^[30]^ a method also recommended by the 6th edition of the WHO Laboratory Manual for the Examination and Processing of Human Semen for the sorting of high-quality sperm.^[7]^ Microfluidic platforms demonstrate significant potential in effectively eliminating erythrocytes and leukocytes from semen samples, while simultaneously providing a targeted approach for sperm selection.^[31,32]^ However, the associated costs and the technical skills required of operating personnel should not be disregarded. Swim-up methods and density gradient centrifugation (DGC) are currently commonly employed for selecting superior sperm in cases of sparse sperm or single sperm cryopreservation in humans.^[33]^ DGC or simplified SLC can separate morphologically normal, DNA-intact motile sperm from the total sperm population, thereby excluding immature or senescent sperm.^[34]^ SLC have been reported to remove contaminating cells and cellular debris in the epididymis of postmortem animals for the selection and preservation of high-quality sperm, exhibiting advantages in maintaining sperm morphology, membrane and DNA integrity.^[35,36]^

Discontinuous DGC has been reported to decrease the rates of DNA fragmentation and sperm malformation.^[5]^ Phillips et al treated a canine blood/sperm admixture with 4 DGC media to separate viable, motile sperm from nonviable sperm and RBCs.^[2]^ Nevertheless, Percoll was determined to compromise sperm membranes and has been discontinued in human clinical use due to endotoxin contamination.^[37,38]^ In our study, we employed the SpermGrad™ and IVF sperm medium diluted proportionally for colloid centrifugation, confirming the efficacy of SLC in removing RBCs from semen.

Short-term abstinence from sperm retrieval has been reported to improve sperm quality in patients with cryptozoospermia, or severe oligozoospermia as well as to increase clinical pregnancy rates and improve embryo quality in intracytoplasmic sperm injection.^[39,40]^ A notable reduction in DFI has been reported following 3 to 4 hours of abstinence.^[41]^ However, there is currently no clear consensus on the optimal duration of short-term sexual abstinence.^[42]^ To minimize the effects of stale bleeding on sperm and to preserve a greater quantity of sperm preoperatively, a 4-hour abstinence was implemented under the condition of ensuring no further bleeding risk, resulting in a significant reduction of RBC in semen and allowing patients to preserve a larger number of motile sperm before formal treatment. The limitations of this research include the absence of a comparison regarding the impact of different abstinence durations on the concentration of RBC and sperm, as well as the lack of functional assessments and morphological evaluations of sperm obtained through a brief abstinence in patients with HS. Furthermore, the optimal abstinence interval for patients with HS remains to be determined in future studies.

## 5. Conclusion

HS considerably affects sperm functionality and the effectiveness of cryopreservation. The treatment of HS may have implications for fertility, especially in individuals with reproductive system tumors. The integration of SLC with a brief abstinence period (2–5 hours) for semen collection has demonstrated its efficacy in eliminating RBCs from semen, providing a feasible approach for autologous semen cryopreservation in clinical settings for patients with HS.

## Author contributions

**Conceptualization:** Yuan Liu, Yang Xian, Fuping Li.

**Data curation:** Bo Liu, Wenrui Zhao, Lijuan Ying, Jinyan Xu, Xuefeng Luo, Chen Luo.

**Funding acquisition:** Fuping Li.

**Writing – original draft:** Yuan Liu.

**Writing – review & editing:** Yang Xian, Fuping Li.
